# Evaluating health claim assessment skills of parents with preschool children: A cross-sectional study using Informed Health Choices Claim Evaluation Tool

**DOI:** 10.7189/jogh.13.04156

**Published:** 2023-11-03

**Authors:** Ivan Pivac, Joško Markić, Tina Poklepović Peričić, Diana Aranza, Ana Marušić

**Affiliations:** 1 University of Split School of Medicine, Split, Croatia; 2 Department of Pediatrics, University Hospital of Split, Split, Croatia; 3 Study of Dental Medicine, University of Split School of Medicine, Split, Croatia; 4 University Department of Health Studies, University of Split, Split, Croatia; 5 Center for Evidence-based Medicine, University of Split School of Medicine, Split, Croatia; 6 Department for Research in Biomedicine and Health, University of Split School of Medicine, Split, Croatia

## Abstract

**Background:**

Health literacy is a global problem and is particularly relevant when making health care decisions about small children. We analysed how parents of preschool children assess health claims and explored the predictors of their assessment skill.

**Methods:**

We conducted a survey with questions from the Claim Evaluation Tools (CET) database, part of the Informed Health Choices (IHC) project, in ten paediatric primary care practices of the Split-Dalmatia County Health Center, Split, Croatia, from 1 February to 31 March 2023. Eligible participants were parents accompanying preschool-aged children for check-ups. We also collected data on parents’ and children’s demographic and health characteristics (including the presence of any chronic illness in the child), visits to paediatric emergency service, hospitalisations, vaccination status, the presence of chronic illness of parents or relatives, and whether parents had to made treatment decisions for themselves and/or their family member.

**Results:**

Overall, 402 parents of preschool children (median age 35 years (interquartile range (IQR) = 31.0-38.3)) had a median IHC CET test score of 10.0 (IQR = 8.0-11.0) out of 12 questions. The multiple regression analysis showed that female gender, higher level of education, being employed, and having a history of a visit to paediatric emergency service were significant predictors of the test score, explaining 21.9% of the variance.

**Conclusions:**

Parents of preschool children have a very good ability for critical assessment of health-related statements in a complex health care system and an environment of generally unsatisfactory health literacy. Further studies should explore how parents understand health claims in different geographical, socio-economic and cultural setting, and explore educational interventions to increase critical thinking abilities and informed decision-making, especially among fathers, unemployed parents and those with lower levels of education.

In recent years, the Internet and social media have become prominent channels for acquiring knowledge about health conditions, self-care practices, and disease prevention across all age groups [1,2], which then influence health behaviour and health care utilisation [3]. However, spreading health information over media outlets has also contributed to the growing epidemic of misinformation, or “infodemic”, as termed by the World Health Organization (WHO) [4]. The proliferation of conspiracy theories, propaganda, and unverified scientific claims related to various topics has made it a global challenge for the public to access and discern reliable information [5]. Furthermore, many media outlets and health care providers simply tell people what to do without enabling them to critically assess health-related information. Merely receiving information is not enough; it must be presented and understood in a manner that does not imply the existence of a “correct” or “incorrect” choice [6].

Health literacy encompasses the individual skills necessary for making appropriate health choices [7] and involves a dynamic interaction between the demands of health care systems and individuals’ skills [8]. It is estimated that half of the population in developed countries possesses inadequate health literacy levels, and more among vulnerable groups and populations in developing countries [8-10]. Low health literacy is also common in health systems that have transitioned from controlled to market economy, like in post-communist countries such as Croatia, where a recent large study showed the level of health literacy to be between problematic and adequate, with most problems in assessing health information on health risks in the media [11]. Health literacy plays a vital role in connecting socio-economic and demographic differences with health outcomes [12], as low health literacy is closely associated with social determinants of health and contributes to disparities, especially among vulnerable populations [13]. Numerous studies demonstrated that deficiencies in health literacy lead to poor health outcomes, inequalities, and increased health care costs [9,14].

Considering the significant expectations imposed on parents when choosing the appropriate treatment for their children within complex health care systems, health literacy emerges as an important consideration in paediatric health care [15]. Many parents encounter difficulties with health literacy, with one out of every four experiencing low health literacy, significantly impacting their capacity to use health information when making decisions concerning their child’s well-being [16,17]. Higher parental health literacy is associated with favourable health behaviours in children, including improved diets, consistent dental hygiene, and increased physical activity [18]. However, inadequate health literacy leads to diminished awareness of prenatal genetic screening and translates into elevated emergency visits and hospitalisations for children with chronic conditions, as well as reduced comprehension of vaccine and new-born screening materials [16,19]. Inadequate parental health literacy extends to unfamiliarity with weight-based medication dosing and reduced breastfeeding rates [19]. For asthma, lower health literacy levels in parents are associated with poorer disease control and increased health care utilisation [20]. Higher parental health literacy corresponds to a nearly 30% longer progression time from chronic kidney disease to dialysis [21] and is related to lower blood glucose levels and HbA1c values in children with diabetes [22]. Parental health literacy interplay with children’s body weight is also evident, as low literacy increases the likelihood of abnormal body weight [23,24].

In response to numerous studies highlighting inadequate levels of health literacy in populations, the European Commission, as well as governments and national agencies in other countries such as the United States, China, and Australia, have developed national strategies and objectives aimed at health literacy promotion for various age groups [8,25,26]. There is also the Informed Health Choices (IHC) project, launched in 2012, with the goal of improving people’s critical thinking abilities and informed decision-making [27,28]. The IHC group, an internationally diverse and interdisciplinary team with expertise spanning methodologies, health services research, medicine, public health, epidemiology, education, communication, and journalism, has developed and evaluated IHC tools to empower individuals in assessing health-related information effectively [28]. The main tools are: 1) Key Concepts, 2) learning materials, and 3) Claim Evaluation Tools (CET) – a bank of multiple-choice questions [27,29]. Key Concepts comprise a list of concepts that serve as a foundation for developing learning materials to help individuals understand and apply these concepts when encountering claims about health interventions and making health decisions [30,31].

Given the absence of prior research on parental awareness of the Key Concepts introduced by the IHC group among parents of preschool children, we aimed to investigate the ability of parents with preschool children to critically evaluate scientific claims. Previous research on IHC Key Concepts has not thoroughly explored how various factors, such as the presence of chronic illnesses in participants and their family members, prior treatment decision-making experiences, number of children, demographic and anthropometric characteristics of the children, vaccination status, frequency of hospitalisations, and visits to the emergency department, impact the success of answering multiple-choice questions from the CET database. Our primary objective was to determine the level of health literacy among parents of children up to 7 years of age and to assess the extent to which demographic characteristics are linked to their health literacy level.

## Methods

### Study design

This was a cross-sectional, questionnaire-based study conducted in 10 paediatric primary care practices of Split-Dalmatia County Health Center in the city of Split, Croatia, from 1 February to 31 March 2023.

### Ethical principles

The research was conducted in accordance with the principles of the Helsinki Declaration [32], following all applicable guidelines aimed at ensuring proper implementation and the safety of individuals participating in the scientific research. Data collection was carried out in fully anonymous fashion, so the General Data Protection Regulation was not relevant. The Ethics Committee of the Split-Dalmatia County Health Center (protocol code: 053-01/22-01/041; date of approval: 22 September 2022) and the Ethics Committee of the University of Split School of Medicine (protocol code: 003-08/22-03/0003; date of approval: 19 December 2022) approved the study.

### Participants

The participants were parents who came with their preschool-aged children for a check-up at a paediatric primary care practice. Considering that there are 14 paediatric primary care practices in the city of Split responsible for the care of approximately 11 800 preschool children, a sample size of 373 was estimated [33] for confidence level of 95% and 5% margin of error. The exclusion criteria were: 1) refusal to participate in the research; 2) having a child aged 7 years or older; 3) visiting the clinic independently or accompanying more than one child; 4) having the questionnaire completed by the other parent of the same child. We randomly selected 10 out of 14 paediatric primary care practices in the city of Split, using a random number generator in Microsoft Excel spreadsheet software, version 16.0 (Microsoft Corporation, Redmond, WA, USA) and sent requests for consent to conduct the research to the respective physicians – clinic leaders. We provided them with comprehensive information about the study and the protocol and, upon their approval, gave them a paper-and-pen questionnaire to distribute to parents in the clinic waiting room. They were specifically instructed to inform the parents that participating in the survey was voluntary and they should not be in the same room with the parents when they filled in the questionnaire. Survey participants were informed in writing about the study purpose, their role in the study, the benefits of participation, and the absence of any risks. Participants were assured that their involvement was voluntary, and that the questionnaire was fully anonymous.

### Questionnaire

The questionnaire had two parts (**Online Supplementary Document** ). The first part collected demographic characteristics of the participants, including age, gender, number of children, educational level, and employment status. It also contained questions related to their child: age, gender, height and weight, presence of any chronic illness in the child, visits to the paediatric emergency service (Pediatric Emergency Department), hospitalisations, and vaccination status. Additionally, the questionnaire collected data on chronic illnesses of the participants and their first- or second-degree relatives, as well as information on whether they have ever made treatment decisions for themselves and/or their family members. Participants were required to answer all questions, except for those regarding the height and weight of their child.

The second part of the questionnaire included questions from the CET database compiled by the IHC Group. The latest version from 2022 contains 49 Key Concepts divided into three categories: claims, comparisons, and choices. They also form the basis for the Claim Evaluation Tools item bank – a set of questions that can be used to assess individuals’ ability to apply specific Key Concepts [31]. We used the Croatian version of the CET, previously validated on high school students [34]. We used the version of the test consisting of 12 questions which can be made available with the author’s permission and consent [34]. The items from the CET item bank are not publicly available [28], but access can be obtained from the IHC project leaders on request. The **Online Supplementary Document** contains the first part of the questionnaire and the list of Key Concepts addressed by the questions selected for this study.

### Statistical analysis

The collected data were coded and entered into a Microsoft Excel spreadsheet version 16.0 (Microsoft Corporation, Redmond, WA, USA). The participants’ reported age was categorised into four categories: 18-24, 25-34, 35-44, and 45-54 years. The children accompanying them to the clinic were categorised by age as infants (0 to <1 year), toddlers/young preschoolers (1 to <3 years), or preschoolers (3 to <7 years). Body mass index (BMI) was calculated using the formula weight/height^2^ (kg/m^2^). Using the child’s gender, age, and the WHO’s standard curves, z-scores were calculated for height, weight, and BMI [35]. According to the WHO classification for children based on the cut off values of z-scores for BMI [36] the following categories were defined: underweight (<-2.0), normal weight (-2.0 to 1.0), overweight (>1.0), obese (>2.0), and severely obese (>3.0).

We used IBM SPSS Statistics, version 23.0 (IBM Corp., Armonk, NY, USA) for data analysis. We presented categorical variables using absolute numbers and percentages and ordinal variables using medians and interquartile ranges (IQRs). We employed the Cronbach’s alpha coefficient of reliability to assess the internal consistency of the items in our measurement instrument. We examined the association between demographic variables and the test scores using Mann-Whitney test for two independent variables, Kruskal-Wallis test for more than two independent variables, and Spearman’s correlation test for ordinal variables. The association between test scores and categorical variables was assessed using the χ^2^ test, and Mann-Whitney test was used for the association with ordinal variables. The independent variables, including gender, education, employment status of the participants, making a treatment decision regarding oneself/family member or their child, nutritional status of the child, and the visit to the Pediatric Emergency Department, all exhibited significant impacts on individual test performance in separate statistical tests. These variables were subsequently incorporated into an Ordinary Least Squares (OLS) multiple regression analysis. The primary objective of this analysis was twofold: first, to investigate their combined multivariate influence on the overall CET test score, serving as the dependent variable, and second, to discern the specific contribution of each variable to the overall test score. We examined the correlation of independent variables included in the multiple regression analysis using Pearson correlation. The level of statistical significance was set at *P* < 0.05.

## RESULTS

The questionnaire was accepted by all 430 parents of preschool children to whom it was offered. We analysed data from 402 participants (93.4%) who completed the questionnaire (**Table 1**). Each paediatric primary care practice had a median of 45 (IQR = 25-51) participants, ranging from 13 to 66 participants. The age of the respondents ranged from 19.0 to 51.0 years, with a median age of 35.0 years (IQR = 31.0-38.3), and the majority were women (80.1%). About half (n = 235 (58.5%)) had at least three years of higher education, with the highest proportion having a master’s degree (n = 170 (42.3%)). Most (n = 317 (78.9%)) of parents were employed at a permanent position. The children had a median age of 3.50 (IQR = 1.82-5.17) years, ranging from 0.08 to 6.99 years.

**Table 1:** Informed Health Choices test score distribution (median, interquartile range) according to the demographic characteristics of study participants (n = 402)

Variable	n (%)	Test score*	*P*-value
**Age in years**			0.107†
18-24	10 (2.5)	5.0 (3.0-10.3)	
25-34	184 (45.8)	10.0 (8.0-11.0)	
35-44	197 (49.0)	10.0 (8.0-11.0)	
45-54	11 (2.7)	8.0 (8.0-11.0)	
**Gender**			<0.001‡
Women	332 (80.1)	10.0 (8.0-11.0)	
Men	80 (19.9)	8.0 (6.0-10.0)	
**Number of children**			0.450†
1	137 (34.1)	10.0 (8.0-11.0)	
2	192 (47.8)	10.0 (8.0-11.0)	
≥3	73 (18.2)	9.0 (7.0-10.5)	
**Education**			<0.001†
Secondary school or lower	167 (41.5)	9.0 (7.0-10.0)	
Bachelor’s degree	57 (14.2)	10.0 (8.0-11.0)	
Master’s level	170 (42.3)	10.0 (9.0-11.0)	
PhD	8 (2.0)	11.0 (10.0-11.8)	
**Employment status**			0.007‡
Unemployed	85 (21.1)	9.0 (7.0-10.3)	
Employed	317 (78.9)	10.0 (8.0-11.0)	
**Chronic disease**			0.060‡
Yes	44 (10.9)	10.0 (9.0-11.0)	
No	358 (89.1)	10.0 (8.0-11.0)	
**Chronic disease in first- and/or second-line relative**			0.051‡
Yes	74 (18.4)	10.0 (9.0-11.0)	
No	328 (81.6)	10.0 (8.0-11.0)	
**Ever making a decision regarding treatment for oneself or a family member**			<0.001‡
Yes	106 (26.4)	10.0 (9.0-11.0)	
No	296 (73.6)	9.0 (8.0-11.0)	
**Ever making a decision regarding treatment for one’s own child**			<0.001‡
Yes	99 (24.6)	10.0 (9.0-11.0)	
No	303 (75.4)	9.0 (8.0-11.0)	

*Range from 0 (minimum) to 12 (maximum).

†Spearman’s correlation test.

‡Mann-Whitney test.

On the test consisting of 12 questions from the IHC CET database, parents had a median score of 10.0 (IQR = 8.0-11.0) correct answers. The Cronbach’s alpha coefficient of reliability was 0.72, indicating moderate reliability of the measurement instrument.

We observed no differences in overall test scores with respect to the participants’ age (**Table 1**). However, mothers achieved significantly higher average scores (median = 10.0 (IQR = 8.0-11.0)) compared to fathers (median = 8.0 (IQR = 6.0-10.0)). We found statistically significant differences between mothers and fathers when analysing the percentages of correct answers for individual questions. Mothers provided more accurate answers for 9 questions, except for questions 4, 8, and 12, where we observed no differences between the groups (Key Concepts: “No comparison needed!”, “More is better!” and “Dissimilar expectations”, respectively) (**Online Supplementary Document**). There was no difference in the overall test scores based on the number of children reported by a parent.

However, the level of education and employment status significantly influenced test performance (**Table 1**). Individuals with a secondary school or lower education had the lowest average scores (median 9.0, IQR = 7.0-10.0). Bachelor’s and master’s degree holders provided better test results, while individuals with a doctoral degree had the highest average median scores of 11.0 (IQR = 10.0-11.8). We observed statistically significant differences in test scores based on the level of education for all questions except for question 9 (Key Concept: “As advertised!” Competing interests may result in misleading claims). We also found statistically significant association between employment status and test performance, with employed individuals having the highest test score in comparison to unemployed individuals.

Regarding participants’ own chronic non-communicable diseases, hypothyroidism (6.0%), other autoimmune endocrine disorders (1.5%), and autoimmune rheumatic diseases (1.0%) were reported most frequently. As for the most common chronic diseases among their closest relatives, type 2 diabetes mellitus (8.2%), cardiovascular diseases (4.2%), asthma (2.2%), and thyroid disorders (2.0%) were most frequent. However, no differences in test scores were observed based on whether the participants themselves or their family members had a chronic illness (**Table 1**). Participants who had ever made decisions regarding treatment for themselves or their family members and those who made decisions about the treatment of their child achieved higher overall test scores (**Table 1**).

Regarding the overall test scores according to the demographic characteristics of the participants’ preschool children, we found that the age and gender of the children were not associated with the test scores (**Table 2**).

**Table 2:** Total Informed Health Choices test scores (median, interquartile range) distribution according to the demographic characteristics of study participants’ children (n = 402)

Variable	n (%)	Test score*	*P*-value
**Age in years**			0.776†
<1	33 (8.2)	10.0 (7.5-11.0)	
1-2	122 (30.3)	10.0 (8.0-11.0)	
3-6	247 (61.4)	10.0 (8.0-11.0)	
**Gender**			0.482‡
Women	184 (45.8)	10.0 (8.0-11.0)	
Man	218 (54.2)	10.0 (8.0-11.0)	
**Nutritional status (n = 338 valid answers)**			0.008§
Underweight	31 (9.2)	10.0 (7.0-11.0)	
Normal weight	222 (65.7)	10.0 (8.0-11.0)	
Overweight	49 (14.5)	9.0 (8.0-10.5)	
Obese	20 (5.9)	8.0 (5.3-10.0)	
Severely obese	16 (4.7)	9.0 (5.5-10.8)	
**Chronic disease**			0.557‡
Yes	25 (6.2)	10.0 (8.0-11.0)	
No	377 (93.8)	10.0 (8.0-11.0)	
**Previous visits to the Pediatric Emergency Department**			<0.001‡
Yes	268 (66.7)	10.0 (8.0-11.0)	
No	134 (33.3)	9.0 (6.0-11.0)	
**Previous hospitalisation**			0.425‡
Yes	98 (24.4)	10.0 (8.0-11.0)	
No	304 (75.6)	10.0 (8.0-11.0)	
**Vaccinated according to the national vaccination schedule**			0.317§
Yes	274 (68.2)	10.0 (8.0-11.0)	
Partially	111 (27.6)	9.0 (8.0-11.0)	
No	17 (4.2)	10.0 (6.5-10.0)	

*Range from 0 (minimum) to 12 (maximum).

†Spearman’s correlation test.

‡Mann-Whitney test.

§Kruskal-Wallis test.

Regarding questions about the height and weight of the child, 338 (84.1%) participants answered both questions. From these data, age- and gender-standardised z-scores were calculated. The average z-scores were 1.0 (IQR = 0.0-2.1) for height, 0.7 (IQR = 0.0-1.4) for weight, and 0.1 (IQR = -0.8-1.0) for BMI. Parents of children with normal nutritional status achieved the highest median test score of 10.0 (IQR = 8.0-11.0), followed by parents of underweight children, children with overweight, and severe obesity, while parents of obese children achieved the lowest score.

The most common chronic diseases of children as reported by the parents were allergic disorders, including atopic dermatitis (2.0%), respiratory disorders such as asthma and bronchitis (1.7%), and neurological disorders (1.0%). Similar to the chronic illnesses of the participants themselves and their family members, the presence of a chronic illness in children was not associated with better test performance.

Parents whose preschool child had visited the Pediatric Emergency Department at least once achieved a higher overall test score (median = 10.0 (IQR = 8.0-11.0)) compared to those whose children had not used it (median = 9.0 (IQR = 6.0-11.0)). The median number of visits to Pediatric Emergency Department for those who had gone at least once was 2.0 (IQR = 1.0-3.0). There were no significant differences in test scores regarding the child’s vaccination status or a history of previous hospitalisation.

**Table 3** presents the distribution of correct answers regarding the IHC Key Concepts in individual questions. Individual questions were answered correctly by a median of 73.7% of the participants (IQR = 69.9%-84.3%). Questions 1 (63.7%) and 4 (67.7%) had the fewest correct answers. These questions assess the understanding of the key concepts “No need to compare!” and “100% certain!” On the other hand, questions 10 and 9, which refer to the key concepts “It worked for me!” and “As advertised!”, had the highest percentage of correct answers (88.6% and 87.1%, respectively) (**Online Supplementary Document**).

**Table 3:** The percentage of study participants correctly answering IHC test questions with different Informed Health Choices Key Concepts (n = 402)

Question	Key Concept*	No. (%)
1	100% safe!	272 (67.7)
2	It works like this!	283 (70.4)
3	Associated with!	287 (71.4)
4	No comparison needed!	256 (63.7)
5	A study shows!	302 (75.1)
6	Old is better!	280 (69.7)
7	New is better!	344 (85.6)
8	More is better!	323 (80.3)
9	As advertised!	350 (87.1)
10	It worked for me!	356 (88.6)
11	Few people or events.	291 (72.4)
12	Dissimilar expectations.	309 (76.9)

*Explanations of the Key Concepts covered by the test used in our study are presented in the **Online Supplementary Document**.

Multiple regression analysis revealed that significant predictors were gender, education, employment status, and a history of visiting the Pediatric Emergency Department, explaining 21.9% of the variance in the overall test score (**Table 4**). The variables regarding making a treatment decision or child’s nutritional status did not show a statistically significant correlation with the test results. However, they exhibited significant correlation with some of the other independent variables in the model (Table S3 in the **Online Supplementary Document**), indicating that their potential influence on the overall results is captured through the other significant variables.

**Table 4:** Multiple regression analysis for the Informed Health Choices total test score*

	Unstandardised coefficient		Standardised coefficient		
**Variable**	**B**	**Standard error**	**Beta**	**T**	***P*-value**	
**(Constant)**	9.52	1.06		9.01	<0.001	
Gender	-1.20	0.33	-0.18	-3.70	<0.001	
Education	0.79	0.14	0.29	5.67	<0.001	
Employment status	0.65	0.32	0.10	2.02	0.044	
Decision: oneself/family member	-0.14	0.43	-0.02	-0.32	0.746	
Decision: one’s own child	-0.45	0.44	-0.07	-1.04	0.301	
Child’s nutritional status	-0.18	0.11	-0.08	-1.65	0.100	
Visit to the Pediatric Emergency Department	-0.95	0.27	-0.17	-3.50	<0.001	

*Ordinary Least Squares multiple regression analysis, R^2^ = 0.235, Adjusted R^2^ = 0.219.

## DISCUSSION

In this study, parents in Croatia who had brought their preschool children for a paediatric check-up demonstrated a high level of understanding of health claims and the effects of health interventions, with over half of the participants showing comprehension of at least 10 out of the 12 analysed Key Concepts. The study was conducted as part of the IHC project, which aims to improve critical health literacy and promote informed health decisions [34,37,38]. In the context of complex health care systems and therapeutic regimens, it is important to ensure effective disease management and achieve positive health outcomes, especially when it comes to parents caring for their children’s health [17]. However, to date, only a single study has assessed parents’ ability to critically evaluate health claims using questions from the Claim Evaluation Tools database developed within the IHC project, conducted in Uganda [39].

The notable similarity in Cronbach's alpha values between our study involving parents (0.72) and study with high school students (0.73) [34] underscores the strength and consistent measurement quality of the test across different participant groups and reaffirms the test’s reliability and suitability for evaluating the IHC Key Concepts in diverse populations.

Regarding the understanding of Key IHC Concepts, this study confirmed that parents have a very good grasp of and apply these concepts, demonstrating their ability to evaluate health claims and treatment effects. The median test result of 83.3% correctly answered questions was higher compared to the reported results of a Norwegian study involving over 600 participants aged 18 years and older, who scored an average of 54.7% correct answers on a 9-item test [40]. Furthermore, high school students in Croatia achieved an average score of 8.4 (70.0%) on the same test used in our study [34]. In the study of parents of children aged 10-12 in Uganda [39], an educational podcast improved the test score from 52.6% to 67.8%, correct answers.

Davies et al. [38] established pass thresholds (i.e. threshold levels of concept application) and mastery thresholds (i.e. full mastery of the concepts) for IHC tests designed for parents and elementary school children. The passing threshold was set at 54.2% for the 24-question multiple-choice test and at 61.6% for the 18-question test, while the mastery threshold necessitated a minimum score of 83.3% on both evaluated tests [38]. In our study, more than three-quarters (79.4%) of the participants achieved a higher score than both established pass thresholds, meaning they obtained at least 66.7% correct answers, while more than half (54.5%) achieved the master level of comprehension of Key Concepts.

The questions “It worked for me!” and “As advertised!” had the highest percentage of correct answers, indicating that the participants were aware that conclusions should not be based on personal experiences and anecdotes, and that conflicting interests often lead to deceptive claims [41]. In turn, the questions “No need for comparison!” and “100% certain!” had the lowest percentage of correct answers, demonstrating parents are not aware that although dramatic treatment effects are easily noticeable without fair comparisons, such effects are extremely rare. Instead, it is important to conduct fair comparisons for treatments with no significant effects to determine their safety and usefulness [42]. Furthermore, we found that parents tend to overestimate treatment benefits and neglect potential harms, which can result in wasting time and money on ineffective treatments that may cause harm [42].

Our study confirmed that the educational status of the participants had the most significant influence on their understanding of health claims. The parents holding doctoral degrees achieved the highest scores, followed by those with master’s degrees and bachelor’s degrees, while individuals with completed primary or secondary education demonstrated the lowest understanding of Key Concepts. This is consistent with the Norwegian IHC study, where multiple regression analysis showed that, along with younger age and Internet access, higher educational status has the greatest impact on successful comprehension of Key Concepts [40]. However, even the parents with completed primary or secondary education in our study demonstrated an understanding of Key Concepts that exceeded both previously defined thresholds for passing [38].

It is assumed that health literacy lies on the pathway between education and health [43]. Long-term studies have clearly confirmed that educational attainment represents a powerful social determinant affecting health [44]. Following the social gradient for education, limited health literacy further deepens socioeconomic inequalities in health. While lower levels of health literacy are often observed in individuals with lower education, even highly educated individuals can exhibit low health literacy skills [43]. Besides the duration of education, the quality and type of educational programs also have a significant impact on the level of health literacy [45]. Aranza et al. [34] have confirmed that students from health and language-focused high schools have a better understanding of Key Concepts compared to students from other high schools. However, Dahlgren et al. [40] did not find a difference in the success rate of answering questions from the IHC CET database between adults who are health care professionals and those who are not. In our study, we did not compare participants based on the type of school they attended.

Furthermore, mothers in our study demonstrated a significantly higher ability to interpret health-related assertions and treatment effects compared to fathers. The enhanced health literacy among women in this study is consistent with previous research findings [44]. The disparity in health literacy between men and women may be linked to traditional gender roles, where women often assume the responsibility of caring for ill family members and children. In our study, more than four-fifths of parents who brought their children for paediatric check-ups were mothers. Additionally, prior studies have substantiated that women are more prone to reporting health issues and utilising medical services than men [44,46]. Consequently, women use health care services more frequently, which affords them greater opportunities for knowledge acquisition and, as a result, a higher level of health literacy in comparison to men [46].

Our study underscores a significant correlation between parental employment status and health literacy. Specifically, we discovered that unemployed parents demonstrate lower levels of health literacy compared to consistently employed counterparts. This pattern corresponds with earlier research by Svendsen et al. [47] which examined the Danish general population, highlighting inadequate health literacy among those receiving unemployment benefits, social assistance, or employment and support allowance, in contrast to those employed in diverse sectors. Moreover, an assessment of the connection between oral health literacy and parental sociodemographic attributes emphasised that unemployed parents exhibit lower levels of oral health literacy than their employed counterparts, alongside other contributing factors [48]. The significance of employment status is further supported by a systematic review, which revealed that mothers engaged in informal employment face elevated risks of suboptimal antenatal care utilisation and child nutritional status [49]. Additionally, the importance of employment status is underlined by the findings that employed mothers, despite time constraints, prioritize enhancing the “quality” of their time [50]. This emphasis is evident in their continued engagement in activities fostering children's development, while allocating less time to potentially hindering activities [50].

We found no significant difference in the critical assessment of health-related statements among parents with or without having their child ever hospitalised. However, parents of children who had visited the Pediatric Emergency Department at least once achieved significantly better results on the test. This contradicts the findings that patients with lower health literacy tend to perceive a higher urgency for medical treatment, are considerably less informed about treatment options, and are more likely to visit the emergency department [51]. A systematic review found evidence confirming the association between low health literacy and increased emergency department utilization in parents of children with asthma, but did not establish a connection between low health literacy and emergency department utilisation in the general paediatric population [52]. The distinct outcomes observed in our study could potentially stem from the effective information conveyed to parents by the medical personnel in the Pediatric Emergency Department regarding health, illnesses, and the impacts of treatments. For certain patients, the emergency department represents their only contact with the health care system, and the stay in the emergency department can provide a “teachable moment” when the patient is receptive to new information. It has been shown that patients and their caregivers demonstrate increased attention and interest during emergency department visits, which provides an opportunity for patient education [53].

Although individual statistical testing revealed a correlation between parents’ test success and the z-score of the child’s BMI, the subsequent multiple regression analysis indicated that this association did not attain statistical significance. Instead, the children’s nutritional status correlated with the level of parental education, confirming the intricate and indirect nature of the relationship between parental health literacy and the child’s body mass index. Nevertheless, a systematic literature review highlighted that parental health literacy is a crucial factor contributing to the growing obesity epidemic [54]. The concerning fact that many parents with low health literacy may not be aware of their child’s overweight status [53] aligns with our findings, where 5% of parents were unaware of their child’s weight, and over 15% were unaware of their child’s height. It has been demonstrated that educational interventions for parents with low literacy reduce childhood obesity up to the age of 18 months, but this effect diminishes by the age of 24 months [55].

The strength of our study lies in the use of a validated measuring instrument. Unlike most other health literacy assessment tools, this validated test, developed as part of the IHC project, is based on objective evaluation, and its results from various studies are comparable [30,31,34]. It can be applied in different educational settings as a self-assessment tool for evaluating the impact of educational interventions, as well as in cross-sectional studies of health literacy. However, one of our study's limitations is that it was conducted in urban clinics with voluntary participation, potentially resulting in an over-representation of participants with higher educational and intellectual levels. Nearly 95% of the parents in our study fell within the 25-44 age range, restricting the generalisability of our findings to that specific age group. While this limitation may imply that our results could misrepresent the target population, conducting the study in ten different paediatric practices enhanced its representativeness. Furthermore, the data we collected relied on self-reports from the participants, which may introduce subjectivity or inaccuracies in assessments. To minimise the prevalence of imprecise data, answering questions about the child’s weight and height was not mandatory. Although our study showed no difference among parents when analysing health-related statements, regardless of whether they or their family members have chronic illnesses, further research is warranted, particularly considering that patients tend to underreport chronic conditions [56]. Finally, despite including several of the participants’ demographic characteristics in this study, these variables accounted for 22% of the variance of the test results, suggesting that other unexplored factors could have influenced the outcomes.

## CONCLUSIONS

We found that parents, accompanying their preschool children to paediatric check-ups, possess a very good ability for critical assessment of health-related statements in a complex health care system and unsatisfactory health literacy at the level of general population [11]. Parents with higher education, women, employed individuals, and parents whose children have visited the Pediatric Emergency Department exhibit the best skills in understanding Key Concepts about treatment claims. This is the first study conducted within the framework of the IHC project that focuses on the population of parents with children up to 7 years old, providing valuable insight into the complex relationship between health literacy, demographic characteristics of parents and their children, and health outcomes. By exploring the role of health literacy in decision-making concerning the health of preschool-aged children, this study contributes to the advancement of health care practices and the improvement of health outcomes for a vulnerable population of small children and their parents. Further research is needed to determine how parents understand health claims in different geographical, socio-economic and cultural setting, and to explore educational interventions to increase critical thinking abilities and informed decision-making.

## Additional material


Online Supplementary Document
