# Supplementary material for: Evaluating health claim assessment skills of parents with preschool children: A cross-sectional study using Informed Health Choices Claim Evaluation Tool
Source: J Glob Health. 2023 Nov 3;13:04156. doi: 10.7189/jogh.13.04156 (PMC10622117; doi:10.7189/jogh.13.04156)
Supplement: Online Supplementary Document [file jogh-13-04156-s001.pdf]

Questionnaire

Table S1. Demographic data

1. Your age (in years):	
2. Your gender:	A) Female<br>B) Male
3. How many children do you have?	A) 1<br>B) 2<br>C) 3 or more
4. Your education:	A) Secondary school or lower<br>B) Bachelor's degree<br>C) Masters level<br>D) PhD
5. Your employment status:	A) Unemployed<br>B) Employed
6. Do you suffer from any chronic diseases? If yes, please specify which one/s?	A) Yes<br>B) No
7. Does any of your 1st and/or 2nd relatives suffer from any chronic diseases? If yes, please specify which one/s?	A) Yes<br>B) No
8. Have you ever made a decision regarding medical treatment for yourself or a family member?	A) Yes<br>B) No
9. Have you ever made a decision regarding medical treatment for your child?	A) Yes<br>B) No
10. Your child's age (in years):	
11. Your child's gender:	A) Female<br>B) Male
12. What is the weight of your child rounded to one decimal place (kg)?*	
13. What is the height of your child rounded to one decimal place (cm)?*	
14. Does your child suffer from any chronic diseases? If yes, please specify which one/s?	A) Yes<br>B) No

15.	Has your child ever been admitted to the Pediatric Emergency Department? If yes, please specify how many times?	A) Yes<br>B) No
16.	Has your child ever been hospitalized? If yes, please specify how many times?	A) Yes<br>B) No
17.	Has your child been vaccinated according to the Croatian vaccination schedule?	A) Yes<br>B) Partially<br>C) No

*Answering the question is not mandatory

Table S2. Key concepts for questions in the study IHC test*

MCQ Question	IHC Key Concept
1	*100% safe!*<br>Treatments can cause harms as well as benefits
2	*It works like this!*<br>Beliefs alone about how treatments work are not reliable predictors of the presence or size of effects.
3	*Associated with!*<br>An ‘outcome’ may be associated with a treatment, but not caused by it.
4	*No comparison needed!*<br>Identifying effects of treatments depends on making comparisons.
5	*A study shows!*<br>The results of one study considered in isolation can be misleading.
6	*Old is better!*<br>Widely used treatments or those that have been used for decades are not necessarily beneficial or safe.
7	*New is better!*<br>Treatments that are new or technologically impressive may not be better than available alternatives.
8	*More is better!*<br>Increasing the amount of a treatment does not necessarily increase its benefits and may cause harm.
9	*As advertised!*<br>Competing interests may result in misleading claims.
10	*It worked for me!*<br>Personal experiences or anecdotes alone are an unreliable basis for most claims.
11	*Few people or events*<br>Small studies may be misleading.
12	*Dissimilar expectations*<br>If possible, people should not know which of the treatments being compared they are receiving.

* The items from the Claim Evaluation Tools item bank are not publicly available (https://www.informedhealthchoices.org/wp-content/uploads/2023/02/Manual-tailoring-your-own-questionnaire_2022.docx) but access can be obtained from the IHC project leaders on request. Table S2 contains the list of Key Concepts of the questions selected for the test in this study.

Table S3. Pearson correlation of independent variables from multiple regression analysis

Variable	Decision: oneself/family member		Decision: one's own child		Child's nutritional status	
	**R**	***P*-value**	**R**	***P*-value**	**R**	***P*-value**
**Gender**	-0.04	0.411	0.03	0.623	0.07	0.175
**Education**	-0.18	<0.001	-0.12	0.014	-0.19	<0.001
**Employment status**	-0.08	0.134	-0.03	0.585	-0.06	0.262
**Visit to Paediatric Emergency Department**	0.11	0.025	0.17	<0.001	0.06	0.289
**Child's nutritional status**	0.10	0.067	0.08	0.133	-	-
**Making health decision about own child**	0.69	<0.001	-	-	-	-
